# The Week's Work

**Published:** 1910-04-16

**Authors:** 


### The Week's Work.

### HOSPITAL SCANDALS.

A correspondent forwards us a cutting from a weekly paper referring to certain abuses which are said to exist at a London hospital. These allegations are made the subject of a violently inimical article under the scare heading "Hospital Scandals," and the writer, who does not scruple to sign his article, waxes indignant at the ill-treatment which is meted out to the poor wretches who attend as out-patients. Our correspondent wishes us to reply to that double-column of misguided journalism, and he points out that to those who have any acquaintance with hospital management the allegations are, on the face of them, absurd. One of these allegations, for instance, is that at the Waterloo Hospital out-patients are kept waiting for hours in the wet and cold, drawn up in a queue and guarded like theatre-goers by a couple of policemen, kept outside in the cold and rain and refused admission till it please the authorities to open the doors. Another is that at Great Ormond Street in-patients are kept waiting in the out-patient department "for two hours," while yet a third is that at most hospitals "nurses are drawn from the servant-girl class . . . and the officer who has risen from the ranks is a notorious failure." The inference in this third allegation is sufficiently obvious to need no further comment.

### LOOSE CHARGES AND FIERY WRITING.

We agree with our correspondent that anyone who knows anything about the inside arrangements of hospitals will not stop to consider such charges as these. The allegations are loose and unsatisfactory even to those who have no such knowledge, and yet they have in them the germs for creating much discontent and ill-feeling. The ordinary person who reads this sort of effusion does not stop to ask himself whether or not such charges are true. He does not reflect that there are, and must be, stated hours for out-patients' attendances at all hospitals, and that such hours must be adhered to, not because of questions of red-tape merely, but solely in order to keep the huge machine that every modern hospital represents smoothly going. He does not think that it is no fault of the authorities if such queues hang about the hospital doors hours before the proper time. As a matter of fact, on rainy days the out-patient doors are invariably opened at an earlier hour, even when such opening entails more trouble and expense to the hospital. It is undoubtedly true that isolated cases of hardship may, and often do, arise. Hospital officers are only human, and it is impossible to be certain that every newly made officer is as charitable, as kind, or as considerate as he ought to be. Every effort, of course, is made to ensure that only men who are thus charitable, humane, and considerate, are chosen; but there are times and occasions when the milk of kindness is somewhat curdled in the most considerate of hospital mankind, and it is well that the public should take this into consideration and not blame the hospital for the fault, when it occurs, of an individual. Such ill-advised articles as those our correspondent forwards, articles written without intimate knowledge of facts, without any attempt to discriminate between real abuse and false allegations, written round one specific point, and dragging in a host of hearsay statements, do an immense amount of harm to hospitals. It is not only the hospital that is named that suffers: every institution in the immediate neighbourhood has to pay for the wrath poured on that one specific hospital by the indignation of the ill-informed scribes. A "scandal" costs a large London hospital thousands of pounds in subscriptions and donations: it may cause hundreds of beds to be closed, and thus indirectly damage the very public which the exposure of such matters is supposed to help.

### HOW TO DEAL WITH THEM.

Is the proper manner to deal with such allegations to ignore them? The hospital worker does ignore them: he goes on his way quietly and calmly, unheeding the mud that is flung at him. But the average member of the public does not take matters so easily. He wants to know the truth. What is he to do? Obviously the best way for him is to submit his complaint to the hospital secretary. If there is anything in it an inquiry will promptly be made, and the abuse, if it exists, will be rectified. It is in the interests of every institution that abuse should be eradicated, and the public may rest assured that where efficiency and thoroughness are the watchwords of a hospital, waiting, useless irritation to patients, unkind nurses, officers who are notorious failures, and other anomalies have no place whatever and will not be allowed to exist. Hospital workers can only hope to counteract the mischief done by such scare articles by educating the public, and that again can only be done by making the public acquainted with the work that is actually done in the wards and in each department of the institution. For that reason such books as Mr. Morris's "History of the London Hospital" do an immense amount of good, and for that reason also we would like to see in the popular press descriptive articles on hospital subjects appear more frequently than they do now. But they should be authoritative articles, and the writers should have first-hand knowledge of the subjects they discuss. Where a hospital scandal is of sufficient general interest to attract public attention the daily press should co-operate with the hospital authorities in dealing with the matter. In that way only can the public be taught to discriminate between the really honest charge of inattention and want of consideration, and the far-reachingly mischievous assertion of general abuse and inefficiency which is the main theme of articles such as those our correspondent has forwarded.

### MEDICAL CERTIFICATES.

Considerable interest attaches to the investigation which the Hospital Saturday Fund has just completed into the provision of medical certificates at the various London hospitals. In view of the complications that may arise from accident or disease should the victim claim compensation under the Workmen's Compensation Act, the issue of such certificates is a matter of great importance. With regard to certificates issued to workmen who may use such documents in order to obtain compensation, the official organ of the Saturday Fund states that the recipient ought to be made to pay for them. It seems that all institutions issue such certificates, stating the nature of the disease or injury, and whether or not the patient is able to follow his usual employment. In no case is a charge made unless expressions of professional opinion, other than statements of facts, are required. Where, however, a certificate is required by the employers, by firms, or by solicitors, the usual fee of a guinea is charged. Some hospitals provide facilities for medical men, acting on behalf of insurance companies or employers, to examine patients in the wards, such examination being conducted in the presence of a house officer. The usual custom is for the superintendent to regulate the fee charged by residents for the issue of certificates wherein expressions of professional opinion are involved. Some months ago a case in connection with such custom arose at the Johannesburg Hospital, where the resident medical officer refused to give a certificate unless the usual guinea was paid by the solicitor. Complaint was made to the House Committee, which, we believe, upheld the view of the resident.

### SCHOOL CERTIFICATES.

The matter of school certificates is an important one in view of the fact that an increasing number of school children attend the London hospitals. Since the London County Council began to subsidise certain hospitals conditionally upon the latter treating certain classes of physically defective children, fresh difficulties have arisen in connection with the matter of granting exemption certificates. Some physicians, we know, refuse to grant certificates on principle. They allege, with perfect justice, that the out-patient card is sufficient proof that the child is attending hospital and ought not to go to school, and we have even heard physicians who advised mothers to "have the inspector up for assault" if he dared force the attendance of the child. This is a perfectly logical position, but it is one the justice of which ordinary inspectors fail to see. They insist upon their certificates, and endless trouble and vexation arise not only to the parents, but sometimes also to the surgeon or physician, unless a proper exempting order is granted. It is high time that some concerted action be taken in the matter. The various hospitals ought to discuss the whole matter, as the Saturday Fund suggests, with the large Funds, and acquire all information that is available. With the opinion that it is not fair that insurance companies and employers, whose interests are always protected, should not pay a fair price for "information required" every hospital worker will cordially agree. Where a poor workingman demands a certificate it should be left to the discretion of the officer in charge of the department to issue it or not, provided always that no refusal is permitted in cases where the certificate is required by a patient too poor to pay the usual fee.

### THE DRUG MARKET.

The improvement in the demand for drugs which was noticed last week is maintained, and although only slight is much appreciated in the drug market. Prices on the whole are well maintained. At present there are no signs indicating a probable reduction in the price of ipecacuanha, but few buyers would be willing to pay the high prices asked unless they were in immediate need of the drug. In spite of the small demand, cod-liver oil is slightly dearer again. Santonin is 2s. 3d. per lb. higher, and it is not improbable that the syndicate which appears to have regained control of this article will advance the price again in due course. Not only has the decline in the price of Buchu leaves ceased, but prices have moved in the other direction and are about 3d. per lb. higher; buyers of Buchu leaves should act with caution. Cubebs are fetching very high prices owing to extreme scarcity; oil of cubebs is also dearer in consequence. The advance in the price of saffron continues. At the recent public sale of drugs in Mincing Lane, rhubarb was slightly cheaper. Makers of cocaine are not willing to sell at the reduced price announced last week, and it is not improbable that the price will be advanced.

